# Effects on Respiratory Pressures, Spirometry Biomarkers, and Sports Performance after Inspiratory Muscle Training in a Physically Active Population by Powerbreath^®^: A Systematic Review and Meta-Analysis

**DOI:** 10.3390/biology12010056

**Published:** 2022-12-29

**Authors:** Diego Fernández-Lázaro, Luis A. Corchete, Juan F. García, David Jerves Donoso, Eva Lantarón-Caeiro, Raúl Cobreros Mielgo, Juan Mielgo-Ayuso, David Gallego-Gallego, Jesús Seco-Calvo

**Affiliations:** 1 Department of Cellular Biology, Genetics, Histology and Pharmacology, Faculty of Health Sciences, University of Valladolid, Campus of Soria, 42004 Soria, Spain; 2 Neurobiology Research Group, Faculty of Medicine, University of Valladolid, 47005 Valladolid, Spain; 3 Cancer Research Center, Biomedical Research Institute, 37007 Salamanca, Spain; 4 Department of Mechanical, Informatics and Aerospatiale Engineering, University of Leon, 24071 Leon, Spain; 5 Department of Anatomy and Radiology, Faculty of Health Sciences, University of Valladolid, Campus of Soria, 42004 Soria, Spain; 6 Pneumology Department of Soria University Assistance Complex (CAUSO), Santa Bárbara Hospital, Castille and Leon Health (SACyL), 42003 Soria, Spain; 7 Faculty of Physiotherapy, University of Vigo, Campus A Xunqueira, 36005 Pontevedra, Spain; 8 Clinical Physiotherapy Research Group (FS1), Galicia Sur Health Research Institute (IIS Galicia Sur), SERGAS-UVIGO, Faculty of Physical Therapy, University of Vigo, 36005 Ponteveda, Spain; 9 Institute of Biomedicine (IBIOMED), Physiotherapy Department, University of Leon, Campus de Vegazana, 24071 Leon, Spain; 10 Department of Health Sciences, Faculty of Health Sciences, University of Burgos, 09001 Burgos, Spain; 11 Physiology Department, Faculty of Medicine, Basque Country University, 48900 Leioa, Spain

**Keywords:** inspiratory muscle training, Powerbreath^®^, maximal inspiratory pressure, forced vital capacity, sports performance, exercise, meta-analysis

## Abstract

**Simple Summary:**

There is currently a growing interest in respiratory muscle training in athletes, so we set out to systematically assess with meta-analyses the effects of IMT with PowerBretah^®^ (PwB), a threshold work device for IMT, on respiratory parameters and athletic performance in healthy physically active adults. Eleven studies were included in the systematic review and nine in the meta-analysis. IMT by PwB significantly increased maximal inspiratory pressure (MIP) and substantial improvements in forced vital capacity (FVC) in the results of the meta-analysis, and sports performance was significantly increased. In conclusion, the IMT with PwB would improve respiratory, MIP, FVC, and sports performance.

**Abstract:**

Sports performance in athletes can be limited by respiratory factors, so it is understandable to propose that inspiratory muscle training (IMT) can improve respiratory function and exercise performance. Power-Breathe^®^ (PwB) is a sectorized respiratory muscle training tool that uses a resistive load to train IMT. There is currently a growing interest in respiratory muscle training, so we set out to systematically assess the effects of IMT with PwB on respiratory parameters and athletic performance in physically active, healthy adults. Based on the Preferred Reporting Items for Systematic Reviews and Meta-Analyses (PRISMA) guideline, the Cochrane and PEDro scales to assess methodological quality, effect size using the Rosenthal formula, and the Cochrane tool for estimation of risk of bias, studies searchable in Medline, Web of Science, and Cochrane. In addition, for the performance of the meta-analysis, the documentation and quantification of the heterogeneity in each meta-analysis were directed through the Cochran’s Q test and the I2 statistic; in addition, a publication bias analysis was performed using funnel plots. Of the total of 241 studies identified in the search, 11 studies for the systematic review and nine for the meta-analysis met the exclusion and/or inclusion criteria. IMT, with PwB, showed significant improvements in maximal inspiratory pressure (MIP) and substantial improvements in forced vital capacity (FVC) in the meta-analysis results. Also, sports performance was significantly increased by IMT with PwB. In conclusion, the use of PwB is an IMT tool that improves respiratory and sports performance.

## 1. Introduction

The inspiratory muscles are histologically and functionally human skeletal muscle fibers and, therefore, are capable of being trained, like any muscle of the musculoskeletal system [1]. Inspiratory muscle training (IMT) is a technique used to increase the strength or endurance of the diaphragm and accessory muscles of inspiration [2]. IMT are non-pharmacological interventions that are low cost, easy to apply, safe, and are considered important adjuncts to the treatment of some lung diseases [3]. IMT has demonstrated improvements in functional capacity, health-related quality of life (HRQoL), pulmonary function, and dyspnea in patients with respiratory conditions [4].

Physical performance in athletes may be limited by respiratory factors such as respiratory muscle dysfunction, exercise-induced hypoxemia, or the initiation of the respiratory metabolic reflex mechanism of the respiratory muscles [5]. It is therefore understandable to propose that IMT can improve exercise performance. The adaptations brought about by a correct training program can influence the energy metabolism of the respiratory muscles, increasing their efficiency and leading to a lower oxygen demand with respect to skeletal muscles [2]. Thus, IMT is a work-breathing procedure that has a possible ergogenic effect on physical exercise in untrained [6] and trained [7] individuals.

IMT devices, which perform sectorized training of the respiratory muscles, can be divided into three categories, such as: resistive charge, voluntary isocapnic hyperpnea, and threshold devices. In this sense, Power-Breathe^®^ (PwB) [PowerBreathe International Ltd. Southam, Warwickshire; England UK] (IMT Technologies LTD) is a resistive loading IMT instrument. Thus, the IMT effect is generated by the adaptation of the inspiratory muscles to overcome the resistance generated by the PwB during inspiration [8]. A recent meta-analysis reported significant improvements in maximal oxygen consumption (VO_2_max) and non-significant improvements in plasma lactate concentration, but imputed a publication bias with respect to these two parameters [3]. In addition, Fernández-Lázaro et al. [3] demonstrated only an increase in respiratory pressures in athletes after IMT with PwB but not in other parameters of pulmonary function.

Thus, and due to the growing interest in the IMT, we set out to critically assess the effects of the IMT with PWB on respiratory pressures, the mechanics of respiratory system biomarkers, and athletic performance in physically active, healthy adults. The research question was defined using the PICO model according to the standard methods proposed by the Preferred Reporting Items for Systematic Reviews and Meta-Analyses Guidelines (PRISMA) [9] as follows: Population: physically active healthy adults (without any respiratory condition); Intervention: inspiratory muscle training through the PwB device; Comparison: placebo/control group or pre/post comparison data group; Outcomes: respiratory pressures (maximal inspiratory pressure [MIP]), spirometry biomarkers (forced vital capacity [FVC], maximal voluntary ventilation [MVV], peak inspiratory flow [PIF], peak expiratory flow [PEF]) and sports performance (mean values of repeated-sprint ability [RSAmea], exercise time [ET], multistage fitness test repetitive [MSFT], time trail [TT], sprints performance [RSP], mean values of power in time trial [WTTmean], number of repetitions, maximal power [Max Power]). These parameters were included as outcomes as they are commonly investigated in studies of IMT.

## 2. Methods

### 2.1. Search Strategy

We developed a structured search using Medline (PubMed), Web of Science (WOS), and Scopus for articles published from the database’s inception to 31 August 2022, restricted to the English and Spanish languages. The search strategy contained terms related to TT and the different outcome biomarkers as well as a combination of these with the Medical Subject Headings (MeSH) index and Boolean operators:: (“Powerbreath”) AND (“muscle” OR “inspiratory muscle training” OR “inspiratory muscle strength”) AND (“performance” OR “athletic performance”) AND (“exercise” OR “physical activity” OR “aerobic capacity” OR “resistance”) (“pulmonary function” OR respiratory parameters”) AND random* (inspiratory muscle training AND atere* AND random*). Two reviewers (D.F.-L. and J.S.-C.) independently screened titles and abstracts, and full texts were sourced for relevant articles. Inclusion criteria were independently assessed, and disagreements were resolved by a third reviewer (L.C.). Additional records were obtained through reference lists that included relevant articles.

### 2.2. Selection Criteria

The selection of records was based on the following criteria: (a) physically active, healthy adults with moderate levels of physical activity or sports practice (recreational, amateur, or professional) without any respiratory condition (including individuals with moderate levels of physical activity and excluding animals); (b) studies that assessed the effects of IMT with PwB as the only method of respiratory training; (c) clinical trials, randomized and not randomized trials, and pre-test/post-test design studies (excluding records, editorials, reviews, notes, and any other non-original study); (d) studies that evaluated as outcomes (primary or secondary) any respiratory pressure (MIP), spirometry biomarkers (FVC, MVV, PEF, PIF), and sports performance; (e) studies with clear information on the intervention protocol of IMT with PwB. Records that did not meet the criteria were excluded from this systematic review.

### 2.3. Quality Assessment

We used the critical review form for quantitative studies developed by updated method guidelines for systematic reviews in the Cochrane Collaboration back review group [10] and the PEDro scale for rating the quality of randomized controlled trials [11].

### 2.4. Quantitative Assessment

A statistical analysis was performed to obtain the main descriptive statistics of central tendency (mean) and dispersion (standard deviation) of the methodological variables of the treatment and of the subjects recruited in the articles analyzed. We calculated the effect size of the results obtained with respect to the different study variables using Rosenthal’s formula [12].

### 2.5. Risk of Bias Assessment

The Cochrane risk bias assessment tool [13] was used to evaluate the quality of the literature by two reviewers (D.F.-L., J.S.-C.). It is a specific tool to assess the risk of bias in clinical trials. For this, it includes a description and assessment for each item in the risk of bias, which offers us a final evaluation that determines the quality of the article [13]. The eight major sources of bias (sequence generation; sssignment concealment; personal blinding; evaluator blinding; incomplete tracking; data reporting; publication bias; observer bias) were classified into three grades: “Yes” indicates a low risk of bias, “No” indicates a high risk of bias, and “Unclear” indicates a lack of information or uncertainty about the potential for bias, which were assessed using the Cochrane risk of bias assessment tool.

### 2.6. Meta-Analysis Data Analysis

To carry out the meta-analysis, the methodology used in the study by Fernández-Lázaro et al. [3] was followed. In this way, the heterogeneity calculation was quantified with Cochrane’s I2 statistic. Without heterogeneity, a fixed effect meta-analysis model was used. However, with heterogeneity, a random effect meta-analysis model was used for evaluating tau squared (τ2) by the DerSimonian-Laird method [14]. Regarding the analysis of publication bias, it was performed using funnel plots, whose asymmetry was quantified through Egger’s regression [15]. The process of performing meta-analysis was used with the metaphor package (version 2.1-0) in R (The R Foundation for Statistical Computing, Vienna, Austria).

### 2.7. Data Extraction

Two reviewers (D.F.-L. and J.S.-C.) reviewed and synthesized the data from all selected studies into a comprehensive table using standardized data extraction. Disagreements were resolved by a third reviewer (D.G.G.). Information extracted from the selected studies included: the name of the first author, year of publication, the country in which the study was conducted, sample size, age, height, and sport activity (Table 1). Also, interventions, instruments, outcomes, and results (Table 2) were included in the review.

## 3. Results

### 3.1. Study Selection

**Figure 1:**
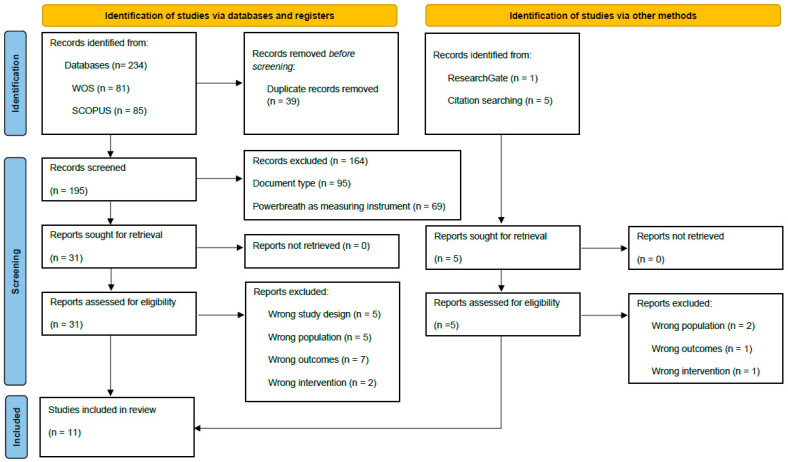
Flow diagram depicting the identification and selection processes of relevant studies according to Preferred Reporting Items for Systematic Reviews and Meta-Analyses (PRISMA) guidelines.

Figure 1 details the process of study selection. The literature search resulted in 240 studies, of which 234 studies were included in the WOS, SCOPUS, and PubMed databases and the remaining six studies were from other sources, such as ResearchGate and reference lists of relevant studies. Thirty-nine duplicates were eliminated, and 195 identified articles were evaluated. Thirty-one manuscripts were included as prospective studies after analysis by title/abstract. After this process, the full text was revised to include a total of 11 records [16,17,18,19,20,21,22,23,24,25,26] in the systematic review.

### 3.2. Characteristics of Participants

Table 1 shows the main characteristics of the participants included in the selected studies and the athletes’ weekly training. The age of the subjects ranged between 18–32 years, and the height range of the participants was 160–182 cm. Five studies included in the review included soccer players [16,18,21,23,24], three studies rugby and basketball players [21,25,26], one study for each sport: field hockey [21], swimming [20], running [17], and cycling [22].

### 3.3. Outcome Evaluation

Table 2 summarizes the contents of the studies contained in this systematic review.

#### 3.3.1. Intervention

The intervention carried out in the selected studies is IMT. Ten of included studies indicate as IMT two sets of 30 breaths (2 × 30) daily [16,18,19,20,21,22,23,24,25,26], only Edwards et al. [17] used 1 × 30 breaths daily. Three studies [17,23,24] included other interventions such as a warm-up [23,24], cardiovascular physical activity [17], and interval training [24] plus IMT. Additionally, 2 studies included IMT with soccer [18] and basketball [25] training.

#### 3.3.2. Respiratory Pressures

Ten studies included in the systematic review evaluated MIP [16,17,18,19,20,21,22,23,24,25]. In 9 of the studies, significant increases (*p* < 0.05) in MIP were obtained in the PwB intervention group with respect to baseline [16,17,18,20,21,22,23,24,25]. In two studies, significant (*p* < 0.05) improvements were described in the intervention group (IG) with respect to the control group (CG). In addition, substantial improvements in MIP were reported in the IG compared to the CG.

#### 3.3.3. Pulmonary Function

Seven studies included in the review evaluated pulmonary function by FVC [16,17,18,20,21,22,26]. In IG compared to baseline, two studies [16,19] showed non-significant decreases, and Vasconcelos et al. [26] showed significant increases (*p* < 0.05). Significant increases (*p* < 0.05) were found in MVV [25], PIF [25] and PEF [26] in the IG compared to baseline.

#### 3.3.4. Sports Performance

All studies [16,17,18,19,20,21,22,23,24,25,26] included in this systematic review evaluated sports performance. Significant [16,18,20,21,23,24,26] (*p* < 0.05) and non-significant [17,19,22,25] (*p* > 0.05) increases in performance have been reported in IG from baseline to study completion. In addition, significant improvements in sports performance in IG compared to CG have been described in the study conducted by Edwards et al. [17] in athletes.

### 3.4. Risk of Bias Assessment

Table 3 shows the analysis of the methodological biases of the studies analyzed in this systematic review by the Cochrane risk bias assessment tool [13]. Two registers presented five biases [17,24], mainly related to allocation concealment, blinding of the personnel, and blinding of the evaluator. Also, three studies presented with four biases [18,19,25] and three studies [20,22,23] presented three biases. Finally, three records showed only two biases [16,21].

### 3.5. Quality Assessment

#### 3.5.1. PEDro Scale

Three studies were considered “good quality” [16,20,23], seven [18,19,21,22,24,25,26] as “regular quality and one [17] as “poor quality” (Table 4). Items six (therapist blinding) and seven (assessor blinding) are the ones in which studies have shown the most deficiencies.

#### 3.5.2. Cochrane’s Assessment of Quality

Five studies were considered such as “good quality” [16,18,19,23,26], 2 [20,21] as “regular quality and 4 [17,22,24,25] as “poor quality” (Table 5). Items 5 (Was the care provider blinded to the intervention?) and 6 (Was the outcome assessor blinded to the intervention?) are the ones in which studies have shown the most deficiencies.

### 3.6. Methodological Variables Assessment

The evaluation of the quantitative analysis of the selected studies can be seen in Table 6. Regarding the sample size, experimental deaths in both the experimental and control groups in the different evaluations (pre-treatment and post-treatment) indicate a low number of dropouts during the interventions. As for the experimental mortality data, all the articles provide data after the treatment period, presenting a mean percentage of 2.05% (SD: 4.71) of dropouts due to different causes. However, none of the studies included a follow-up evaluation.

### 3.7. Effect Size Assessment

Table 7 shows the results of the calculation of the effect size of the selected studies. The effect size offered disparate results depending on the study analyzed. According to this statistical analysis, the treatment had a large effect size (d > 0.8) and thus a greater magnitude of effect. In relation to the improvement of sports performance, Romer et al. [21] (d = 1.81), Tong et al. [24] (d = 0.89), Tong et al. [23] (d = 1.03), and Archiza et al. [16] (d = 1.19) showed a large effect size in their interventions.

For MIP, seven studies showed improvements with a large effect size [16,17,21,22,23,24,25], with an effect size between 1.56 d and 0.95 d. Regarding lung function (FVC), only the study conducted by Romer et al. [21] showed a high effect size (d = 1.29). Vascocelos et al. [26], Salazar-Martínez et al. [22] and Tranchita et al. [25], who obtained significant improvements in FVC, reported results of moderate magnitude (d > 0.5).

### 3.8. Evaluation of the Results of the Studies Included in the Synthesis and Meta-Analysis (n = 9 Included Studies)

#### 3.8.1. Maximal Inspiratory Pressure (*n* = 8 Included Studies)

**Figure 2:**
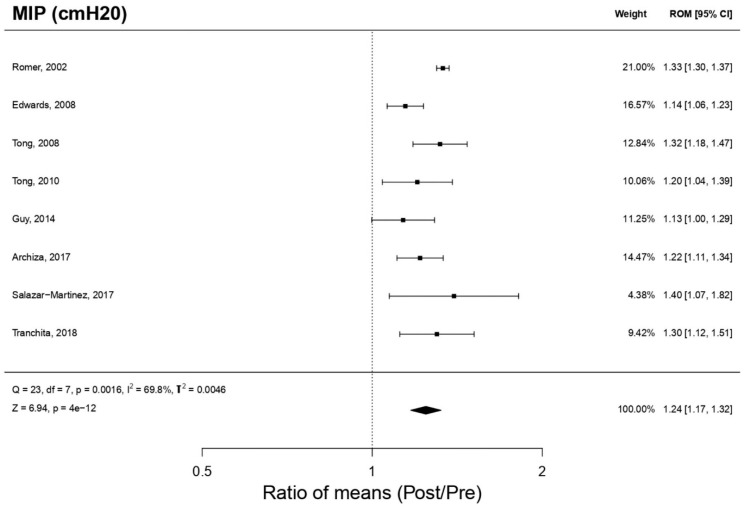
Forest Plot meta-analysis results for Maximal Pressure Inspiratory.

**Figure 3:**
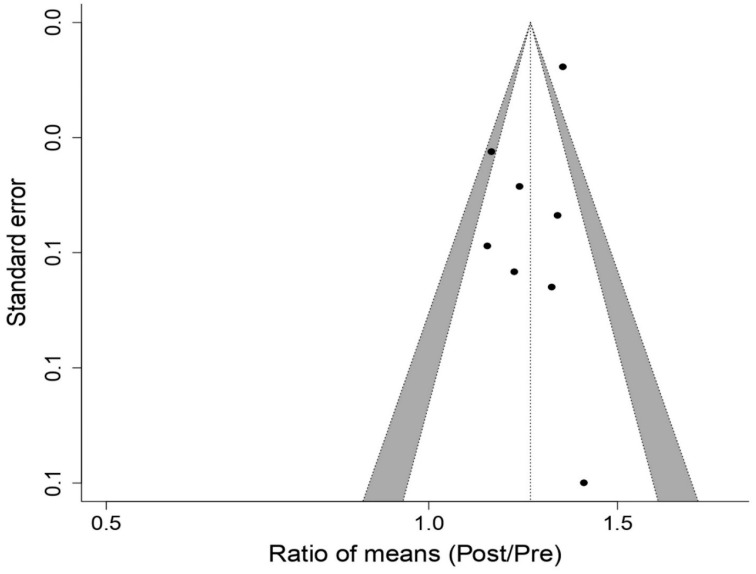
Funnel Plot meta-analysis results for Maximal Pressure Inspiratory.

Figure 2 shows the effect of using the PwB on MIP, a statistically significant increase effect is produced (*p* < 0.05): ROM 1.24; 95% CI, 1.17 to 1.32; Z = 6.94; *p* = 4 × 10^−12^ for the studies [16,17,18,21,22,23,24,25] analyzed in the meta-analysis. The publication bias analysis (Figure 3) for the MIP presented a relatively symmetric funnel plot, without any imputed studies, which could indicate that there is no publication bias, although this asymmetry was not statistically significant ’value of Egger’s *p* = 0.238). Through the “Trim and fill” method, there was no imputation of studies at levels higher than ES and under standard error.

#### 3.8.2. Forced Vital Capacity (*n* = 6 Included Studies)

**Figure 4:**
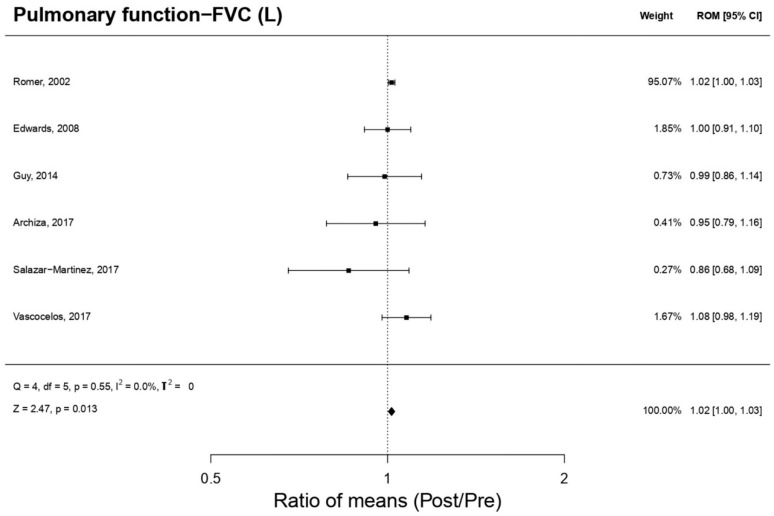
Forest Plot meta-analysis results for Force Vital Capacity.

**Figure 5:**
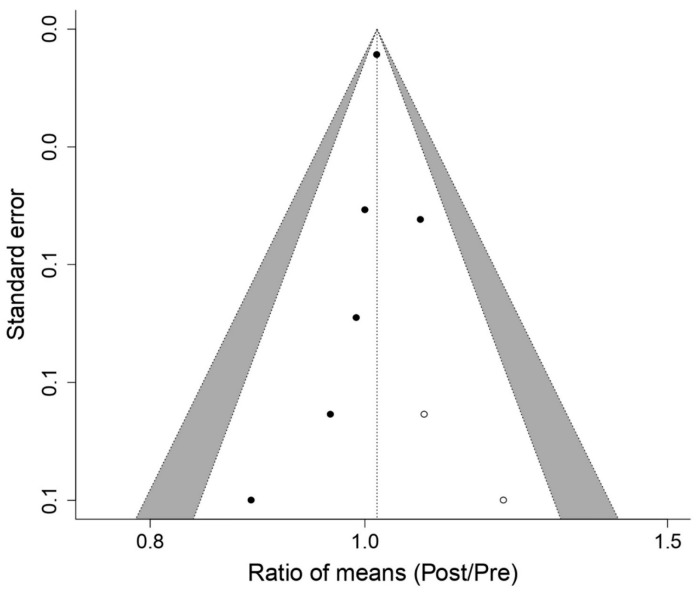
Funnel Plot meta-analysis results for Force Vital Capacity.

Figure 4 shows the effect of using the PwB on FVC, a statistically non-significant increase effect is produced (*p* > 0.05): ROM 1.02; 95% CI, 1.00 to 1.03; Z = 2.47; *p* = 0.013 for the studies [16,17,18,21,22,26] analyzed in the meta-analysis. The publication bias analysis (Figure 5) for the MIP presented a relatively symmetric funnel plot, without 2 imputed studies, which could indicate that there is publication bias, and this asymmetry was not statistically significant ’value of Egger’s *p* = 0.369). Through the “Trim and fill” method, there was imputation of 2 studies at levels higher than ES and under standard error, which could indicate a possible lack of studies at this level.

## 4. Discussion

The aim of this systematic review was to critically evaluate the effects of IMT with PWB on respiratory pressures, mechanics of the respiratory system, biomarkers, and sports performance in physically active, healthy adults. Eleven studies met the pre-specified inclusion/exclusion criteria. Overall, subjects who performed IMT with PwB showed significant improvements in MIP and substantial improvements in FVC in the meta-analysis results. In addition, this systematic review found significant improvements in sports performance [16,18,20,21,23,24,26] and other spirometry biomarkers such as peak inspiratory flow [25] and peak expiratory flow [26]. The energetic commitment of the respiratory muscles (RMs) with respect to the skeletal muscles that develop active physical activity and the fatigue of the RMs are the two main factors that restrict the respiratory function, causing a decrease in respiratory performance [27]. RMs demand a high percentage of cardiac output (15–20%) in situations of vigorous physical activity, which requires a reduction in the availability of oxygen (O_2_) for the skeletal muscle involved in the exercise and therefore produces a notable decrease in performance in these athletes [28,29]. Thus, the high O_2_ needs of RMs during intense exercise conditions generate a competitive demand with respect to active skeletal muscles, which see a significant decrease in the supply of oxygen to their cells, and this causes a decrease in sports performance [30].

During intense and/or prolonged exercise, the appearance of RMs fatigue is possible, following the depletion of its energy substrates [31] and the response of the sympathetic nervous system that activates the respiratory metabolic reflex [3]. The respiratory metabolic reflex involves vasoconstriction that triggers a decrease in blood flow and an increase in the severity of skeletal muscle fatigue, induced by flow and redistributed to preserve respiratory function without compromising RM energy demand [32]. Furthermore, significant decreases in MIP and maximum expiratory pressure (PEM) have been described after long-term and/or intense aerobic physical exercise [33]. However, training the respiratory musculature, particularly IMT, which completes the athlete’s training, attenuates the competition for blood flow, improves tolerance to respiratory fatigue, and increases the efficiency of RMs, all of which have a positive influence on athletic performance [3,34,35].

Our findings indicating benefits in MIP, FVC, and sport performance could be a consequence of adaptations induced by proper IMT program implementation. Especially important could be the improvements in the MIP determined in the meta-analysis, which are also consistent with those reported in other studies [7,36,37,38,39] and in a recent meta-analysis of IMT that found that administering a resistive load of 15% of the MIP achieved significant improvements although they did not reach the pre-established threshold of statistical significance [3]. The substantial improvements in FVC reported in our meta-analysis in athletes are relatively surprising because, in athletes prior to IMT, lung function is close to supraphysiological limits, that is, the values of the main pulmonary ventilation parameters are ≥100% of physiological level [40]. IMT usually induces improvements in FVC in adults with limited lung function [41,42], due to the state of weakness of their respiratory muscles and decreased lung volumes [43,44]. In this sense, IMT in patients with heart failure reduces the magnitude of the respiratory metabolic reflex, which could prevent functional deterioration and MR atrophy and therefore significantly improve lung function [45].

These adaptations to IMT try to modulate homeostatic processes [46] that lead to changes in the oxidative energy metabolism of RMs, gaining in efficiency and causing a lower oxygen demand with respect to skeletal muscles [28,29], tissue remodeling of RMs (hypertrophy of the diaphragm, increase of type II fibers) that increases their strength and functionality [34], and the delay of the respiratory metabolic reflex [3]. Also, PIM´s improvements could also indicate optimization of the neuro-motor control of the respiratory musculature, maintaining the generation of pressure with a lower motor impulse and greater economy of the respiratory musculature [23]. The achievement of these biological adaptations, which involve totally different mechanisms, would be responsible for improvements in sports performance [47,48]. Significant improvements in IG from baseline and compared to CG athletes [17] have been found in the studies included in the systematic review of different sports modalities as soccer [16,18,21,23,24], swimming [20], rugby [21,23,24], basketball [21], and field hockey [21]. 

In addition to serving as adjuvant therapy in pathological states [41,42,45,49], IMT could be used in populations that perform jobs with high physical demands, such as the military, emergency services, or high mountain rescuers, who usually carry backpacks and heavy loads [50]. In addition, these populations usually use masks or respiratory devices, thus increasing respiratory work, which would induce early muscle fatigue and a reduction in work tolerance [51].

Evidence presented in this systematic review and meta-analysis suggests that IMT by PwB device is safe and provides significant improvements in MIP and substantial improvements in FVC and sports performance. The mechanisms of improvement of respiratory biomarkers by IMT could have a multifactorial etiology, mainly the attenuation of the respiratory metabolic reflex and the modulation of respiratory muscle fatigue. However, these results on IMT with PwB in physically active, healthy adults without chronic diseases need to be confirmed.

## Figures and Tables

**Table 1:** Characteristics of participants in the selected studies.

First Author and Year of Publication	n (IG)	n (CG)	Age (Years)	Height (cm)	Sport Activity	Weekly Training Volume of Athletes
Archiza et al., 2017 [16]	N_pre_ = 10 N_post_ = 10	N_pre_ = 8 N_post_ = 8	IG: 22.0 (3.9) CG: 20.1 (2.0)	160.0 (0.1) 160.0 (0.0)	Soccer (p)	20 h × wk^−1^; 60% physical and 40% technical and tactical training
Edwards et al., 2008 [17]	N_pre_ = 8 N_post_ = 8	N_pre_ = 8 N_post_ = 8	NA	180.1 (4.5) 181.3 (5.0)	Running (r)	NA
Guy et al., 2014 [18]	N_pre_ = 24 N_post_ = 21	N_pre_ = 12 N_post_ = 10	IG1: 26.6 (8.2) IG2: 23.9 (6.7) CG: 21.3 (4.9)	182.0 (0.1) 175.0 (0.1) 175.0 (0.1)	Soccer (r)	Twice-weekly sessions for pre-season training
Hart et al., 2001 [19]	N_pre_ = 6 N_post_ = 6	N_pre_ = 6 N_post_ = 6	32.0 (4.8)	NA	NA	NA
Kilding et al., 2016 [20]	N_pre_ = 8 N_post_ = 8	N_pre_ = 8 N_post_ = 8	IG: 19.1 (2.6) CG: 19.0 (2.1)	176.5 (4.0) 180.5 (6.5)	Swimming (p)	NA
Romer et al., 2001 [21]	N_pre_ = 12 N_post_ = 12	N_pre_ = 12 N_post_ = 12	IG: 21.3 (1.1) CG: 20.2 (0.7)	174.0 (0.1) 177.0 (0.1)	Soccer, rugby, field hockey, and basketball (p and/or a)	NA
Salazar-Martínez et al., 2017 [22]	N_pre_ = 8 N_post_ = 8	N_pre_ = 8 N_post_ = 8	IG: 23.4 (2.7) CG: 25.4 (3.2)	180.2 (3.5) 168.8 (5.1)	Cycling (a)	NA
Tong et al., 2008 [23]	N_pre_ = 20 N_post_ = 20	N_pre_ = 10 N_post_ = 10	IG1: 21.3 (0.9) IG2: 21.5 (2.1) CG: 22.0 (2.9)	175.0 (5.4) 174.7 (6.8) 175.0 (5.4)	Soccer & rugby (a)	NA
Tong et al., 2010 [24]	N_pre_ = 9 N_post_ = 9	N_pre_ = 9 N_post_ = 9	IG: 21.1 (1.1) CG: 22.3 (1.0)	172.9 (3.8) 175.6 (4.0)	Soccer & rugby (a)	2–3 h × day^−1^ for 4–5 days × wk^−1^
Tranchita et al., 2018 [25]	N_pre_ = 15 N_post_ = 15	N_pre_ = 14 N_post_ = 14	IG: 21.06 (2.5) CG: 19.0 (2.1)	181.4 (9.9) 181.1 (9.6)	Basketball (a)	8 h × wk^−1^
Vasconcelos et al., 2017 [26]	N_pre_ = 12 N_post_ = 11	N_pre_ = 11 N_post_ = 10	IG: 22.0 (5.0) CG: 18.5 (5.8)	NA	Basketball (p)	NA

Abbreviations: n: sample size; IG: intervention group; CG: control group; Npre: sample size at the baseline; Npost: sample size at the end of the study; IG1: intervention group 1; IG2: intervention group 1; NA: data not available; (p): professional level; (a): amateur level; (r): recreational level; h: hour; wk: week.

**Table 2:** Studies included in the systematic review of the effect of PowerBreath^®^ in physically active, healthy adults.

Study	T	Interventions	Instrument	Outcomes (Units)	Results	
					IG: Changes from Baseline	IG vs. CG
Archiza et al., 2017 [16]	6	IMT (2 × 30) With PwB device	Mouth pressure meter, spirometer	Respiratory pressures MIP (cmH_2_O) Pulmonary function FVC (L) Sports performance RSAmean (s)	↑* MIP ↓ FVC ↑* RSAmean	ND
Edwards et al., 2008 [17]	4	IMT (1 × 30) + Cardiovascular training (CV1: 5 × 1000 m; CV2: 3 × 1600 m; SP: 20 min) With PwB device	Mouth pressure meter, portable ergospirometer	Respiratory pressures MIP (cmH_2_O) Pulmonary function FVC (L) Sports performance ET (s) 1000 m	↑* MIP ↔ FVC ↑ ET	↑* MIP ↔ FVC ↑* ET
Guy et al., 2014 [18]	6	IMT (2 × 30) + pre-season soccer training (2 days per week) With PwB device	Spirometer, chronometer, heart rate monitor, lactate analyzer	Respiratory pressures MIP (cmH_2_O) Pulmonary function-FVC (L) Sports performance-MSFT (m)	↑* MIP ↓FVC ↑* MSFT	ND
Hart et al., 2001 [19]	6	IMT (2 × 30) With PwB device	Mouth pressure meter, spirometer, chronometer	Respiratory pressures MIP (cmH_2_O) Pulmonary function MVV (L/min) Sports performance ET (s)	↑ MIP ↑ MVV ↑ ET	↑ MIP ↑ MVV ↑ ET
Kilding et al., 2016 [20]	6	IMT (2 × 30) With PwB device	Mouth pressure meter, spirometer, lactate analyzer	Respiratory pressures MIP (cmH_2_O) Pulmonary function FVC (L) Sports performance TT 200 m (strokes/min)	↑* MIP ↑ FVC ↑* TT	↑* MIP ↑ FVC ↑ TT
Romer et al., 2001 [21]	6	IMT (2 × 30) With PwB device	Pneumotachograph spirometer, hand-held mouth pressure meter, lactate analyzer.	Respiratory pressures MIP (cmH_2_O) Pulmonary function FVC (L) Sports performance RSP (s)	↑* MIP ↑ FVC ↑* RSP	ND
Salazar-Martínez et al., 2017 [22]	6	IMT (2 × 30) With PwB device	Spirometer, cycloergometer, portable gas analyzer	Respiratory pressures MIP (cmH_2_O) Pulmonary function FVC (L) Sport performance WTTmean (W)	↑* MIP ↑ FVC ↑ WTT	ND
Tong et al., 2008 [23]	6	Warm-up + IMT (2 × 30) With PwB device	Bidirectional gas flow meter, RPE and RPB scales	Respiratory pressures MIP (cmH_2_O) Sports performance (number of repetitions)	↑* MIP ↑* number of repetitions	↑ MIP ↑ number of repetitions
Tong et al., 2010 [24]	6	Warm-up + IMT (2 × 30) + Interval training With PwB device	Differential pressure transducer, portable ergospirometer	Respiratory pressures MIP (cmH_2_O) Sports performance (number of repetitions)	↑* MIP ↑* number of repetitions	↑ MIP ↑ number of repetitions
Tranchita et al., 2018 [25]	4	IMT (2 × 30) With PwB device	Spirometer, Astrand-Rhyming Cycle Ergometer Test	Respiratory pressures MIP (cmH_2_O) Pulmonary function PIF (L/min) Pulmonary function MVV (L/min) Sports performance Max Power (W)	↑* MIP ↑* PIF ↑*MVV ↑ Max Power	ND
Vasconcelos et al., 2017 [26]	4	IMT (1 × 30) With PwB device	Spirometer	Pulmonary function FVC (L) Pulmonary function PEF (L/s)	↑* FVC ↑*PEF	ND

Abbreviations: T: temporality (weeks); IG: intervention group; CG: control group; IMT: inspiratory muscle training; (*n* × 30): 30 dynamic inspiratory efforts twice daily; (1 × 30): 30 dynamic inspiratory efforts once a day; PwB: powerbreathe; CV1: cardiovascular test 1; CV2: cardiovascular test 2; SP: self-paced running; RPE: rate of perceived exertion; RPB: rate of perceived breathlessness; MIP: maximal inspiratory pressure; FVC: forced vital capacity; L: litres; cmH_2_O: centieater of water; RSP: repetitive sprints performance; MVV: maximal voluntary ventilation; min: minutes; ET: exercise time; s: seconds; m: meters; MSFT: multistage fitness test; RSAmean: mean values of repeated-sprint ability WTTmean: mean values of power in time trials; W: watt; PIF: peak inspiratory flow; PEF: peak expiratory flow; ND: not described; ↑ = no significant increase; ↓ = no significant decrease; ↔ = no significant change. ↑* = significant increase; ↓* = significant decrease.

**Table 3:** Cochrane’s assessment of risk of bias [13].

Items	Archiza et al., 2017 [16]	Edwards et al., 2008 [17]	Guy et al., 2014 [18]	Hart et al., 2001 [19]	Kilding et al., 2016 [20]	Romer et al., 2001 [21]	Salazar-Martínez et al., 2017 [22]	Tong et al., 2008 [23]	Tong et al., 2010 [24]	Tranchita et al., 2018 [25]	Vascocelos et al., 2017 [26]
**1**	NO	NO	NO	NO	NO	NO	NO	NO	NO	NO	YES
**2**	YES	NO	NO	NO	NO	NO	NO	NO	NO	NO	YES
**3**	NO	NO	YES	NO	YES	YES	YES	YES	NO	NO	NO
**4**	YES	NO	NO	NO	NO	YES	NO	NO	NO	NO	NO
**5**	YES	YES	YES	YES	YES	YES	YES	YES	YES	YES	YES
**6**	YES	NO	NO	YES	YES	YES	YES	YES	NO	YES	YES
**7**	YES	YES	YES	YES	YES	YES	YES	YES	YES	YES	YES
**8**	YES	YES	YES	YES	YES	YES	YES	YES	YES	YES	YES
**Total**	**2**	**5**	**4**	**4**	**3**	**2**	**3**	**3**	**5**	**4**	**2**

Abbreviations = 1: sequence generation; 2: assignment concealment; 3: personal blinding; 4: evaluator blinding; 5: incomplete tracking; 6: data reporting; 7: publication bias; 8: observer bias; the rating for each item includes the response to a question“, where “Yes” indicates a low risk “f ” bias, “No” indicates a high risk of bias, and “Unclear” indicates a lack of information or uncertainty about the potential for bias; the higher the score, the higher the risk of bias.

**Table 4:** Results of the methodological quality assessment of included studies—PEDro Scale [11].

Items	Archiza et al., 2017 [16]	Edwards et al., 2008 [17]	Guy et al., 2014 [18]	Hart et al., 2001 [19]	Kilding et al., 2016 [20]	Romer et al., 2001 [21]	Salazar-Martínez et al., 2017 [22]	Tong et al., 2008 [23]	Tong et al., 2010 [24]	Tranchita et al., 2018 [25]	Vascocelos et al., 2017 [26]
**1**	1	0	0	0	0	0	1	0	0	0	1
**2**	1	1	1	1	1	1	1	1	1	1	1
**3**	1	0	0	0	0	0	0	0	0	0	1
**4**	1	1	1	1	1	1	1	1	1	1	1
**5**	1	0	1	0	1	1	1	1	0	0	0
**6**	0	0	0	0	0	0	0	0	0	0	0
**7**	0	0	0	0	0	1	0	0	0	0	0
**8**	1	-	1	1	1	0	0	1	0	0	1
**9**	1	-	0	1	0	0	0	1	1	0	0
**10**	1	1	1	1	1	1	1	1	1	1	1
**11**	1	0	0	0	1	0	1	0	0	1	0
**Total**	8	3	5	5	6	5	5	6	4	4	5
**Quality**	G	P	R	R	G	R	R	G	R	R	R

Abbreviations = 1: eligibility criteria specified; 2: random allocation; 3: concealed allocation; 4: groups similar at baseline; 5: subject blinding; 6: therapist blinding; 7: sssessor blinding; 8: less than 15% dropouts; 9: intention-to-treat analysis; 10: between-group statistical comparisons; 11: paint measures and variability data; -: not evaluable; 1: YES; 2: NO; Quality score = total YES score; 9–11: eIllent (E); 6–8: good (G); 4–5: regular (R); <4: poor (P).

**Table 5:** Cochrane’s assessment of quality [10].

Items	Archiza et al., 2017 [16]	Edwards et al., 2008 [17]	Guy et al., 2014 [18]	Hart et al., 2001 19]	Kilding et al., 2016 [20]	Romer et al., 2001 [21]	Salazar-Martínez et al., 2017 [22]	Tong et al., 2008 [23]	Tong et al., 2010 [24]	Tranchita et al., 2018 [25]	Vascocelos et al., 2017 [26]
**1**	NO	NO	NO	NO	NO	NO	NO	NO	NO	NO	YES
**2**	YES	NO	NO	NO	NO	NO	NO	NO	NO	NO	YES
**3**	YES	YES	YES	YES	YES	YES	YES	YES	YES	YES	YES
**4**	YES	NO	YES	NO	YES	YES	YES	YES	NO	NO	NO
**5**	NO	NO	NO	NO	NO	NO	NO	NO	NO	NO	NO
**6**	NO	NO	NO	NO	NO	YES	NO	NO	NO	NO	NO
**7**	YES	YES	YES	YES	YES	YES	YES	YES	YES	YES	YES
**8**	YES	-	YES	YES	YES	-	-	YES	-	-	YES
**9**	YES	-	YES	YES	NO	-	-	YES	-	-	YES
**10**	YES	YES	YES	YES	YES	YES	YES	YES	YES	YES	YES
**11**	YES	-	NO	YES	NO	-	-	YES	-	-	NO
**Total**	8	3	6	6	5	5	4	7	3	3	7
**Quality**	G	P	G	G	R	R	R	G	P	P	G

Abbreviations = 1: Was the method of randomization adequate?; 2: Was the treatment allocation concealed?; 3: Were the groups similar at baseline?; 4: Was the patient blinded to the intervention?; 5: Was the care provider blinded to the intervention?; 6: Was the outcome assessor blinded to the intervention?; 7: Were co-interventions avoided or similar?; 8: Was the compliance acceptable in all groups?; 9: Was the drop-out rate described and acceptable?; 10: Was the timing of the outcome assessment similar in all groups similar?; 11: Did the analysis include an intention-to-treat analysis?; -: Not evaluable; 1: YES; 2: NO; Quality score = total YES score; 9–11 excellent (E); 6–8: good (G); 4–5: regular (R); <4: poor (P).

**Table 6:** Characteristics of the sample and the intervention of selected articles and their quantitative analysis.

Moderators’ Variables	k	Min.	Max.	Mean	SD
**Intervention variables**					
Duration (weeks)	11	4	6	5.45	0.93
Intensity (hours/week)	0	NA	NA	NA	NA
Magnitude (hours/intervention)	0	NA	NA	NA	NA
**Subjects’ variables**					
Age (years)	10	19.05	32	22.47	3.73
**Methodology’s variables**					
SS of the experimental group, at pretreatment	11	6	24	12	5.6
SS of the experimental group, at post-treatment	11	6	21	11.63	5
SS of the experimental group, at follow-up	0	-	-	-	-
SS of the control group, at pretreatment	11	6	15	9.72	2.57
SS of the control group, at post-treatment	11	6	15	9.45	2.42
SS of the control group, at follow-up	0	-	-	-	-
Mortality at post-treatment evaluation (%)	11	0	13.88	2.05	4.71
Mortality at follow-up evaluation (%)	0	-	-	-	-

Abbreviations = SS: simple size; %: percentage; k: number of articles; Min.: minimum range; Max.: maximum range; SD: standard deviation; NA: data not available; -: not calculable.

**Table 7:** Calculation of effect si’e by Rosenthal’s formula.

Study	Outcomes (Units)	M (DT) pre	M (DT) Post	*p*-Value	ES
Archiza et al., 2017 [16]	Respiratory pressures MIP (cmH_2_O)	137 (15.3)	166.5 (17.1)	<0.05	1.51
	Pulmonary function FVC (L)	4.4 (1.0)	4.2 (0.9)	>0.05	0.27
	Sports performance RSAmean (s)	7.9 (0.2)	7.6 (0.2)	<0.05	1.19
Edwards et al., 2008 [17]	Respiratory pressures MIP (cmH_2_O)	148.1 (13.7)	169.5 (9.1)	<0.01	1.56
	Pulmonary function FVC (L)	5.5 (0.4)	5.5 (0.6)	>0.05	0.00
	Sports performance-ET (s) 1000 m	210 (52.2)	205 (53.8)	0.09	0.09
Guy et al., 2014 [18]	Respiratory pressures MIP (cmH_2_O)	134 (24.0)	152 (21.0)	0.002	0.75
	Pulmonary function FVC (L)	5.25 (0.99)	5.19 (0.9)	>0.05	0.06
	Sports performance MSFT (m)	1491 (410)	1666 (460)	0.02	0.42
Hart et al., 2001 [19]	Respiratory pressures MIP (cmH_2_O)	127.8(ND)	143.4 (NA)	0.02	-
	Pulmonary function MVV (L/min)	174 (ND)	186 (NA)	0.65	
	Sports performance ET (s)	848 (ND)	887 (NA)	0.22	
Kilding et al., 2016 [20]	Respiratory pressures MIP (cmH_2_O)	115 (26.0)	NA	<0.01	0.41
	Pulmonary function FVC (L)	5.2 (0.7)		0.60	−0.07
	Sports performance TT 200 m (strokes/min)	43.7 (5.1)		0.02	−0.25
Romer et al., 2001 [21]	Respiratory pressures MIP (cmH_2_O)	130.3 (3.7)	173.8 (6.0)	<0.01	1.29
	Pulmonary function FVC (L)	5.63 (0.09)	5.72 (0.09)	>0.05	1.00
	Sports performance RSP (s)	243.9 (9.2)	227.2 (9.0)	<0.01	1.81
Salazar-Martínez et al., 2017 [22]	Respiratory pressures MIP (cmH_2_O)	119.6 (37.36)	166.91 (42.65)	<0.05	1.26
	Pulmonary function FVC (L)	5.44 (1.14)	4.67 (1.38)	>0.05	0.67
	Sport performance WTTmean (W)	217.25 (49.07)	241.87 (56.01)	0.02	0.50
Tong et al., 2008 [23]	Respiratory pressures MIP (cmH_2_O)	145.1 (19.6)	191.3 (22.2)	<0.05	1.35
	Sports performance (number of repetitions)	37.6 (5.9)	43.7 (6.6)	<0.05	1.03
Tong et al., 2010 [24]	Respiratory pressures MIP (cmH_2_O)	163 (29.8)	195.9 (23.5)	<0.01	1.10
	Sports performance (number of repetitions)	40.3 (5.0)	52.7 (6.4)	<0.05	0.89
Tranchita et al., 2018 [25]	Respiratory pressures MIP (cmH_2_O)	97.75 (23.85)	127.25 (22.12)	<0.001	0.95
	Pulmonary function PIF (L/min)	66.67(23.60)	87.58 (29.88)	0.005	0.77
	Pulmonary function MVV (L/min)	125.50(20.37)	133.83 (25.0)	0.013	0.42
	Sports performance Max Power (W)	158 (34.48)	161 (34.50)	>0.05	0.08
Vasconcelos et al., 2017 [26]	Pulmonary function FVC (L)	4.03 (0.45)	4.34 (0.51)	<0.05	0.68
	Pulmonary function PEF (L/s)	6.73 (1.51)	7.17 (1.58)	<0.05	0.52

Abbreviations = M (SD) pre: mean (standard deviation) at pretreatment; M (SD) post: mean (standard deviation) at post-treatment evaluation; ES: effect size; MIP: Maximum Inspiratory Pressure; FVC: Forced Vital Capacity; MVV: maximal voluntary ventilation; RSAmean: mean performance time; ET: exercise time; MSFT: multistage fitness test; TT: time trail; WTTmean: average watts; PIF: peak inspiratory flow; PEF: peak expiratory flow; NA: data not available. -: not calculable.

## Data Availability

Not applicable.
